# The prevention of chemotherapy induced peripheral neuropathy by concurrent treatment with drugs used for bipolar disease: a retrospective chart analysis in human cancer patients

**DOI:** 10.18632/oncotarget.23467

**Published:** 2017-12-19

**Authors:** Roxanne J. Wadia, Marilyn Stolar, Clarice Grens, Barbara E. Ehrlich, Herta H. Chao

**Affiliations:** ^1^ Department of Internal Medicine, Section of Medical Oncology, Yale School of Medicine, New Haven, Connecticut, USA; ^2^ Yale Comprehensive Cancer Center, Yale School of Medicine, New Haven, Connecticut, USA; ^3^ Yale Center for Analytical Sciences, Yale School of Public Health, New Haven, Connecticut, USA; ^4^ Department of Pharmacology, Yale School of Medicine, New Haven, Connecticut, USA; ^5^ VA Connecticut Healthcare System, West Haven, Connecticut, USA

**Keywords:** chemotherapy, taxane, vinca alkaloid, lithium, valproic acid

## Abstract

Peripheral neuropathy is a major adverse effect in the use of chemotherapeutic drugs. In nearly 50% of patients, chemotherapy induced peripheral neuropathy (CIPN) has been reported as irreversible. With increasing numbers of patients surviving treatment as well as increasing duration of survival after treatment, reducing the side effects of chemotherapy and improving the quality of life has become a major focus of cancer survivorship. Multiple classes of chemotherapeutic drugs including taxanes, platinum agents and vinka alkaloids list peripheral neuropathy as the main dose-limiting side effect of treatment. We previously found that drugs that interfere with the microtubule function, including taxanes and vinca alkaloids, bind to neuronal calcium sensor 1 (NCS1), leading to aberrant calcium signaling. The altered calcium signaling can be mitigated by application of drugs used to treat bipolar disease (e.g., lithium and valproic acid) prior to initiation of chemotherapy. Because pre-treatment with these drugs prevented CIPN in mice treated with taxanes, we sought clinical evidence by performing a retrospective chart review study of the VA electronic health record to see whether or not there would be evidence to support our scientific belief that patients treated with lithium or valproic acid while receiving chemotherapy have a lower risk for development of CIPN than patients who received chemotherapy alone. Our data did provide evidence supporting the belief that treatment with lithium or valproic acid concurrently with chemotherapy was associated with a decreased incidence of developing CIPN.

## INTRODUCTION

Chemotherapy is presently the most common treatment for a broad variety of cancers and is likely to remain as such for the foreseeable future despite the recent progress in cancer immunotherapy. However, chemotherapy induced peripheral neuropathy (CIPN) remains a significant dose-limiting toxicity that can lead to prolonged morbidity and decreased quality of life in a large proportion of patients who receive chemotherapy [1]. It is most commonly associated with the platinum agents, taxanes and vinka alkaloids. CIPN is associated with symptoms that include tingling, numbness, neuropathic pain and ataxia. More recently, in a cohort study of 462 women almost half of the patients continued to report symptoms of CIPN after an average of 5.8 years out from their diagnosis [2]. At this time there are limited treatment options for the treatment of CIPN and no treatment options for the prevention of CIPN [3].

In many cases, the severity of the chemotherapy-related side effects requires a reduction in a patient’s dosing, either in the amount or the timing of the therapeutic drug [4], often reducing the effectiveness of the treatment. If chemotherapy side effects could be precluded or mitigated, patients would be less likely to experience dose reductions, intermittent discontinuations, or termination of a chemotherapy regimen due to toxicity, and potentially would be more likely to receive optimally safe and effective regimens. The post-treatment quality of life for affected patients would also be much improved.

Two classes of drugs that interfere with microtubular function, taxanes and vinca alkaloids, have been used extensively to treat malignant tumors [5, 6] and are frequently used for the treatment of ovarian, breast, lung and prostate cancers. The two drug classes have opposite effects on microtubules. Taxanes such as paclitaxel and docetaxel stabilize microtubules and vinca alkaloids like vincristine and vinorelbine disrupt microtubules. This change of microtubular dynamics in both assembly and disassembly leads to cell cycle arrest and lack of cell division [7–9]. It is this feature of these drugs that makes them effective as chemotherapeutic agents [7–9].

In previous work [10, 11] we identified a novel binding partner for taxanes and vinca alkaloids, neuronal calcium sensor 1 (NCS1), that appears to be a critical component of the pathway leading to the initiation of CIPN. When these chemotherapeutic drugs bind to NCS1, intracellular calcium signaling is altered leading to activation of calpain, a calcium-dependent enzyme [10, 12]. Activated calpain then catalyzes the degradation of a number of proteins, including NCS1 [11, 12], resulting in change in neuronal function. Modification of NCS1 to a calpain-resistant variant protected cells from paclitaxel-induced decreases in calcium signaling [11]. *In vivo* experimental support for this mechanism of neuronal damage is that inhibition of calpain in mice had a protective effect against paclitaxel-induced sensory neuropathy in mice [13]. In a more direct *in vivo* test of the role of NCS1 in the development of CIPN using a model of paclitaxel-treated mice, lithium (Li) and valproic acid (VPA) prevented degradation of NCS1, maintained intracellular calcium signaling, and provided protection from treatment-induced tissue damage [14]. Of note, the anti-neoplastic effects of the treatment were not impacted [14]. These pre-clinical results using cell culture and mouse models suggest that it may be possible in humans to prevent CIPN with Li or VPA concurrent treatment.

The purpose of our study was to perform an analysis of data from a retrospective chart review for patients who had received chemotherapy containing taxanes, focusing on the commonly used chemotherapy agent docetaxel as treatment for solid tumor malignancies in order to add patient-based knowledge to our research regarding the use of concurrent Li or VPA to prevent CIPN. Our analysis indicated that the data support furthering the investigation into the role of Li and VPA and their use in the prevention of CIPN.

## RESULTS

### Summary of cancer location and severity

**Figure 1:**
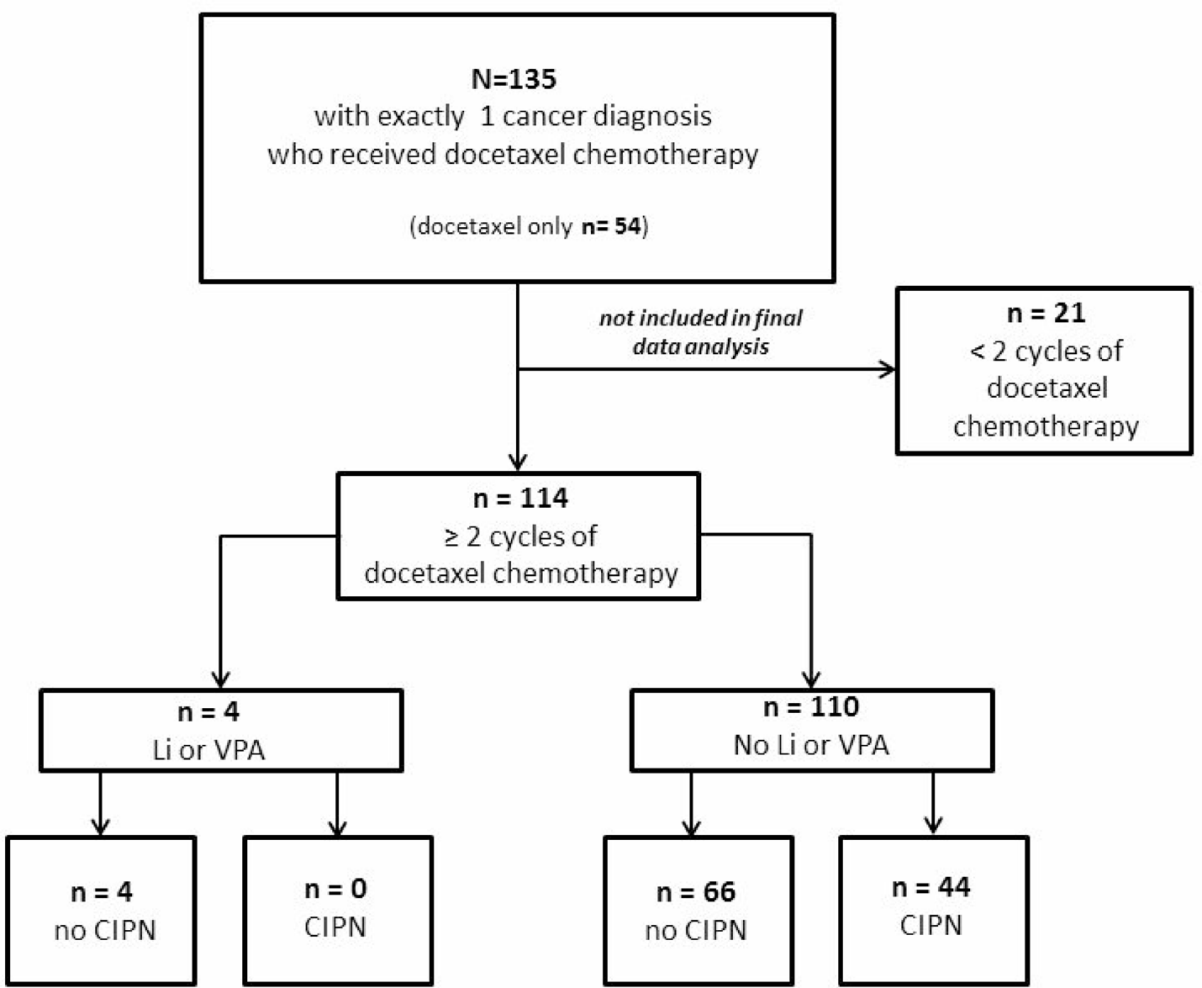
Flow diagram for patient inclusion in the analysis data set

Of the 135 cases that were identified in the cancer registry and pharmacy records, 114 patients were included in the final analysis (Figure 1). 25 (22%) of the cases had lung cancer, 51 (44%) had prostate cancer, 10 (9%) had gastric or esophageal cancer and 22 (19%) had head and neck cancer (Table 1). The remaining 6 patients (5%) had sarcoma, non-melanomatous skin cancer, urothelial carcinoma, or unclassifiable tumors. Stage IV disease at the time of receiving the chemotherapy of interest was diagnosed in 111 (97%) of the patients (Table 1). Pre-existing neuropathy and comorbidities were also captured.

**Table 1:** Baseline characteristics of study population

Variable	All *N* = 114	Li or VPA *N* = 4	No Li or VPA *N* = 110
Age at diagnosis (years) mean (SD)	67.5 (9.8)	70.5 (11.6)	67.4 (9.8)
Primary Malignancy:	*n* (%)	*n* (%)	*n* (%)
Gastro-Esophageal	10 (9)	0	10 (9)
Head and Neck	22 (19)	1 (25)	21 (19)
Lung	25 (22)	1 (25)	24 (22)
Prostate	51 (44)	2 (50)	49 (44)
Sarcoma	2 (2)	0	2 (2)
Skin	1 (1)	0	1 (1)
Urothelial	1 (1)	0	1 (1)
Other	2 (2)	0	2 (2)
Pre-existing neuropathy	20 (18)	1 (25)	19 (17)
Pre-existing diabetes	19 (17)	0	19 (17)
Prior or current heavy alcohol use	42 (37)	1(25)	41 (37)
Current heavy alcohol use	8 (7)	0	8 (7)
Pre-existing substance abuse	9 (8)	0	9 (8)
Pre-existing neurologic disease	20 (18)	1 (25)	19 (17)
Pre-existing psychiatric disease	33 (29)	3 (75)	30 (29)

### Analysis of development of CIPN

Because CIPN is associated with cumulative doses of chemotherapy, it was decided to include only the 114 patients who received at least 2 cycles of treatment (Table 2). Of those, 4 (4%) had concurrent use of Li or VPA and chemotherapy (“*Li or VPA”*) and 110 (96%) did not (“*No Li or VPA”*). Among the 4 patients with concurrent treatment, none (0%) were found to have worsening of prior peripheral neuropathy or to have developed *de novo* CIPN. Among the 110 patients without concurrent treatment, 44 (40%) had worsening of prior peripheral neuropathy or developed *de novo* CIPN.

**Table 2:** Development or worsening of CIPN

*n* (row%)	All			Prior neuropathy no			Prior neuropathy yes		
Li or VPA	CIPN no	CIPN yes	total	CIPN no	CIPN yes	total	CIPN no	CIPN yes	total
**no**	66 (60)	44 (40)	110	59 (65)	32 (35)	91	7 (37)	12 (63)	19
**yes**	4 (100)	0 (0)	4	3 (100)	0 (0)	3	1 (100)	0 (0)	1
total	70 (61)	44 (39)	114	62 (65)	32 (35)	94	8 (40)	12 (60)	20

### Case studies of patients receiving Li or VPA concurrently with chemotherapy

The 4 patients who received treatment with Li or VPA concurrently with chemotherapy are described in the following:

Patient #1 was a male patient who was diagnosed with stage IV lung squamous cell cancer at age 62. He received initial palliative chemotherapy with Carboplatin and Paclitaxel for 5 cycles. Upon progression of his lung cancer his treatment was changed to second line chemotherapy with Docetaxel for 3 cycles. The patient was diagnosed with paranoid schizophrenia in his early twenties and had been receiving chronic treatment with valproic acid and loxapine prior to the diagnosis of lung cancer. His medication compliance was closely monitored by the mental health clinic prior to his cancer diagnosis. While he was undergoing palliative chemotherapy he was staying at a nursing facility where his medications were managed by the pharmacy and nursing teams on a daily basis. Prior to receiving any chemotherapy the patient had baseline mild numbness in his left arm and left hand of unclear etiology. The patient was regularly assessed for any potential worsening or new onset neuropathy throughout his cancer treatment and did not have any changes. He died 11 months after his lung cancer diagnosis.

Patient #2 was a male patient who was diagnosed with stage IV salivary gland carcinoma at age 59. He was initially treated with combination chemotherapy consisting of Carboplatin/5-Fluorouracil/Cetuximab for 2 cycles without response. His chemotherapy was changed to Docetaxel. He was continued on Docetaxel for 4 cycles. He had longstanding history of bipolar disorder for which he had been treated with lithium and quetiapine starting several years prior to his cancer diagnosis. He was maintained on stable doses of lithium and quetiapine throughout his cancer treatment. His compliance with lithium and quetiapine was closely monitored by the mental health clinic. The patient did not develop any signs or symptoms of peripheral neuropathy until his death 18 months after the diagnosis of stage IV cancer.

Patient #3 was a male patient with a longstanding history of bipolar disorder that was treated with aripiprazole and valproic acid and history of chronic renal insufficiency due to past lithium/thioridazine toxicity. As a result of his psychiatric medications he developed tardive dyskinesia. A remote history of a lacunar infarct likely contributed to his movement disorder but he did not have any evidence for peripheral neuropathy as assessed by his neurologist. He was diagnosed at age 73 with Gleason 7 prostate cancer. He received radiation treatment to the prostate bed as initial localized therapy. When he was noted to have rising PSA he was initiated on androgen deprivation therapy with LH-RH agonist injections at age 75. At age 80 he developed castrate-resistant prostate cancer with multiple bone metastases and lung metastases. He received palliative chemotherapy with Docetaxel for total of 8 cycles with transient improvement in his cancer-related symptoms. Throughout his cancer treatment he was maintained on stable doses of aripiprazole and valproic acid and did not develop any signs or symptoms of peripheral neuropathy until he died at age 81.

Patient #4 was a male patient who was diagnosed with Gleason 9 prostate cancer at age 77. His initial staging scans were negative for metastatic spread of the prostate cancer. He received radiation treatment to the prostate and pelvis as localized therapy and was started on androgen deprivation with LH-RH agonist injections. While on androgen deprivation he developed castrate resistant prostate cancer with new bone metastases at age 78. He received palliative radiation to a painful thoracic vertebral spine metastasis in T4 and started secondary hormonal manipulation with oral Abiraterone and Prednisone and monthly infusions of zolendronic acid for skeletal protection. While on Abiraterone/Prednisone he sustained a minor stroke that was complicated by a partial seizure that progressed into a grand mal seizure. His seizure was treated initially treated with levetiracetam but due to a neutropenic reaction his anti-epileptic therapy was changed to valproic acid. His medications were managed by his daughter with regular refills of his prescriptions documented in the pharmacy records. Upon further progression of his prostate cancer on Abiraterone/Prednisone he was started on palliative chemotherapy with Docetaxel at age 79 and received total of 4 cycles of Docetaxel. The patient did not develop any type of neuropathy throughout chemotherapy until he died at age 80.

### Bayesian analysis

**Figure 2:**
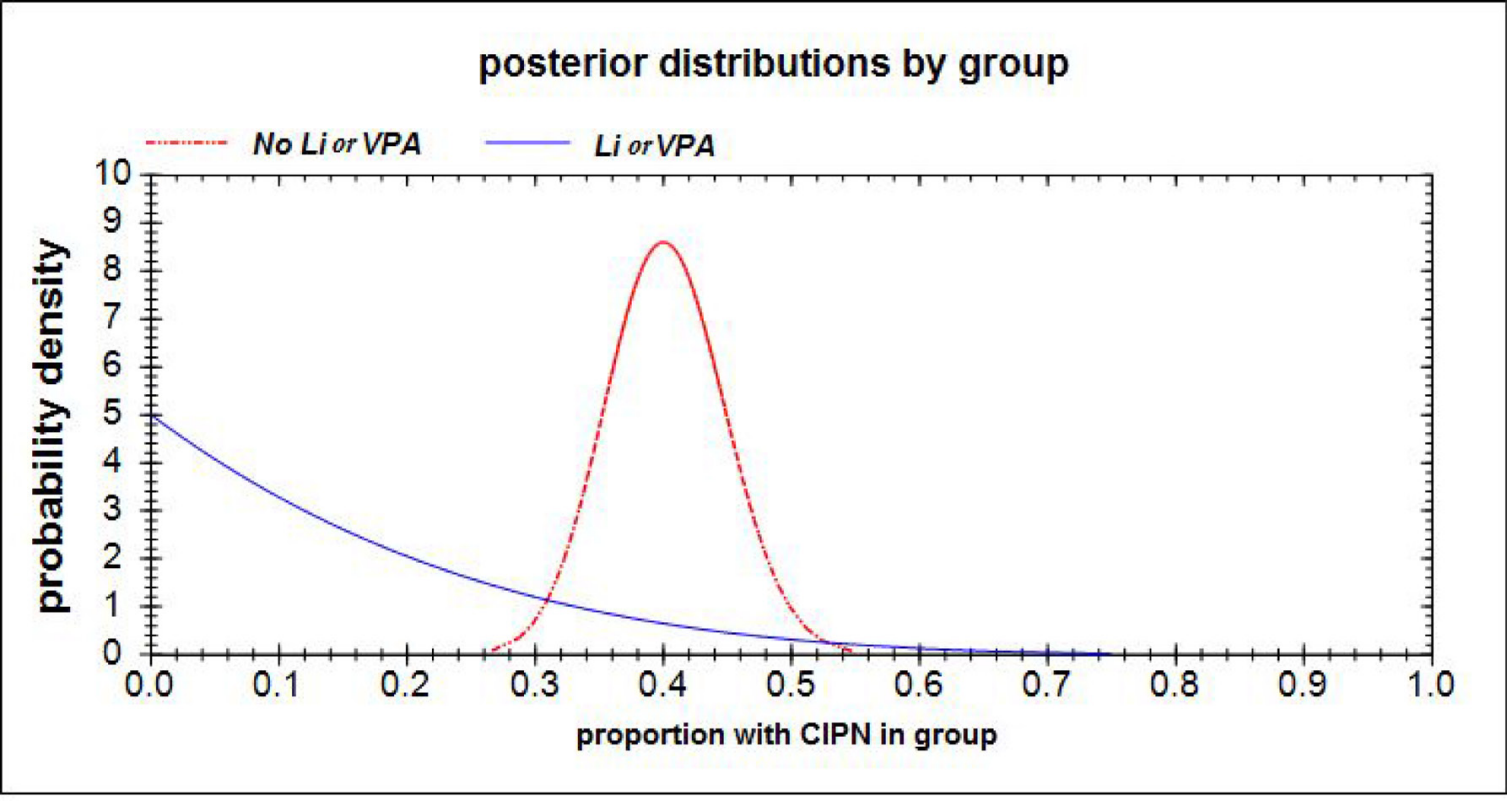
Based upon a non-informative prior probability distribution Beta(1, 1) for both *p*_*1*_ (the proportion of CIPN in the “*No Li or VPA”* group) and *p*_*2*_ (the proportion of CIPN in the *“Li or VPA”* group), and new information from the chart review of 0 CIPN of 4 in Li + VPA group and 44 CIPN of 100 in the *“No Li or VPA”* group, the respective posterior probability distributions for the group CIPN proportions are *p*_*1*_∼Beta(1, 5) and *p*_*2*_∼Beta(45, 67) These posterior distributions for *p*_*1*_ and *p*_*2*_ are represented by the red and blue curves, respectively.

**Figure 3:**
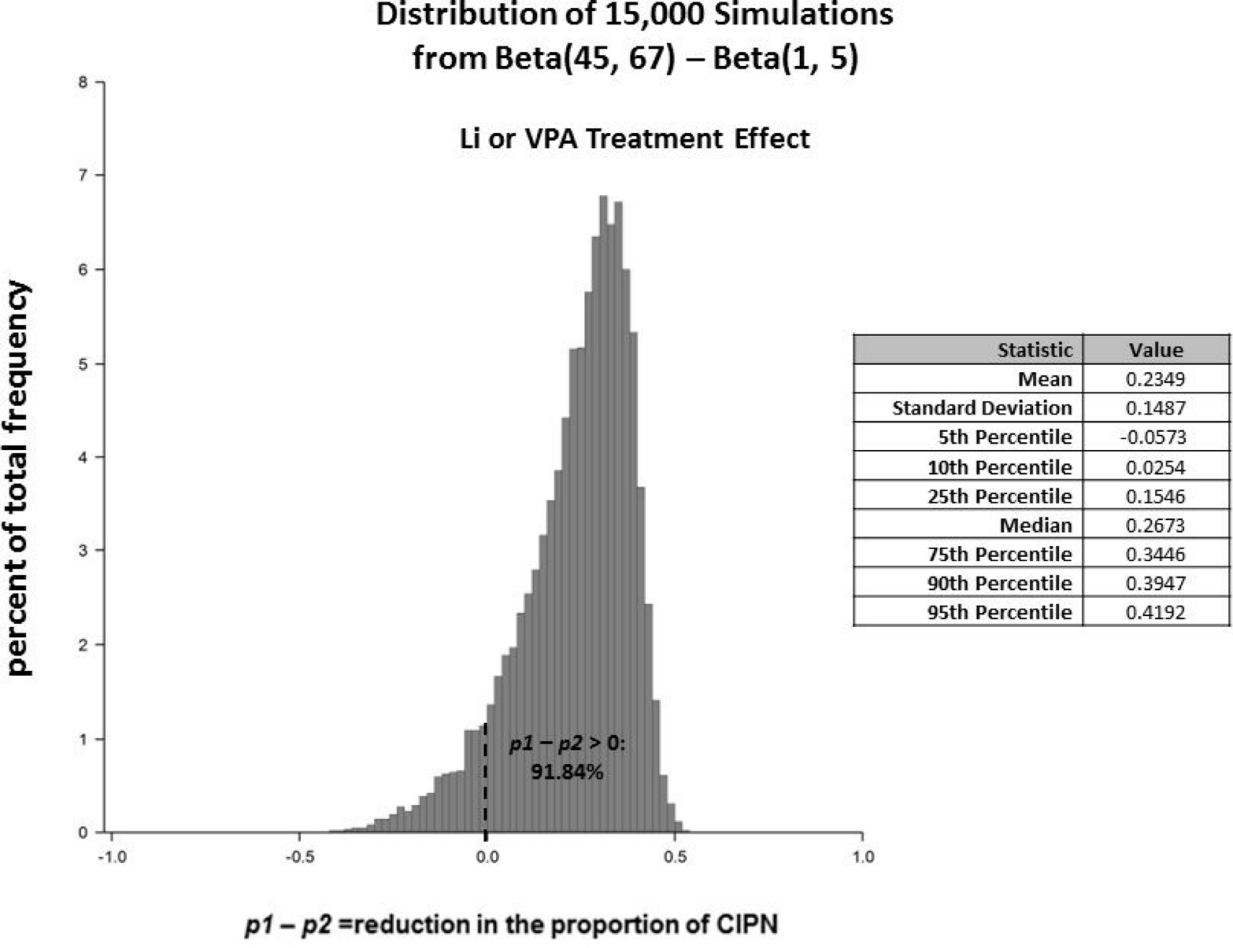
The posterior probability distribution for the *“Li or VPA”* treatment effect is *p*_*2*_ – *p*_*1*_ ∼ Beta(45, 67)-Beta(1, 5) This probability distribution was generated from 15,000 simulated random draws and is shown along with summary statistics. The area under the curve to the right of *p*_*2*_ *– p*_*1*_ > 0 represents the probability that the “*Li or VPA”* group has lower risk of CIPN than the “*No Li or VPA”* group.

**Figure 4:**
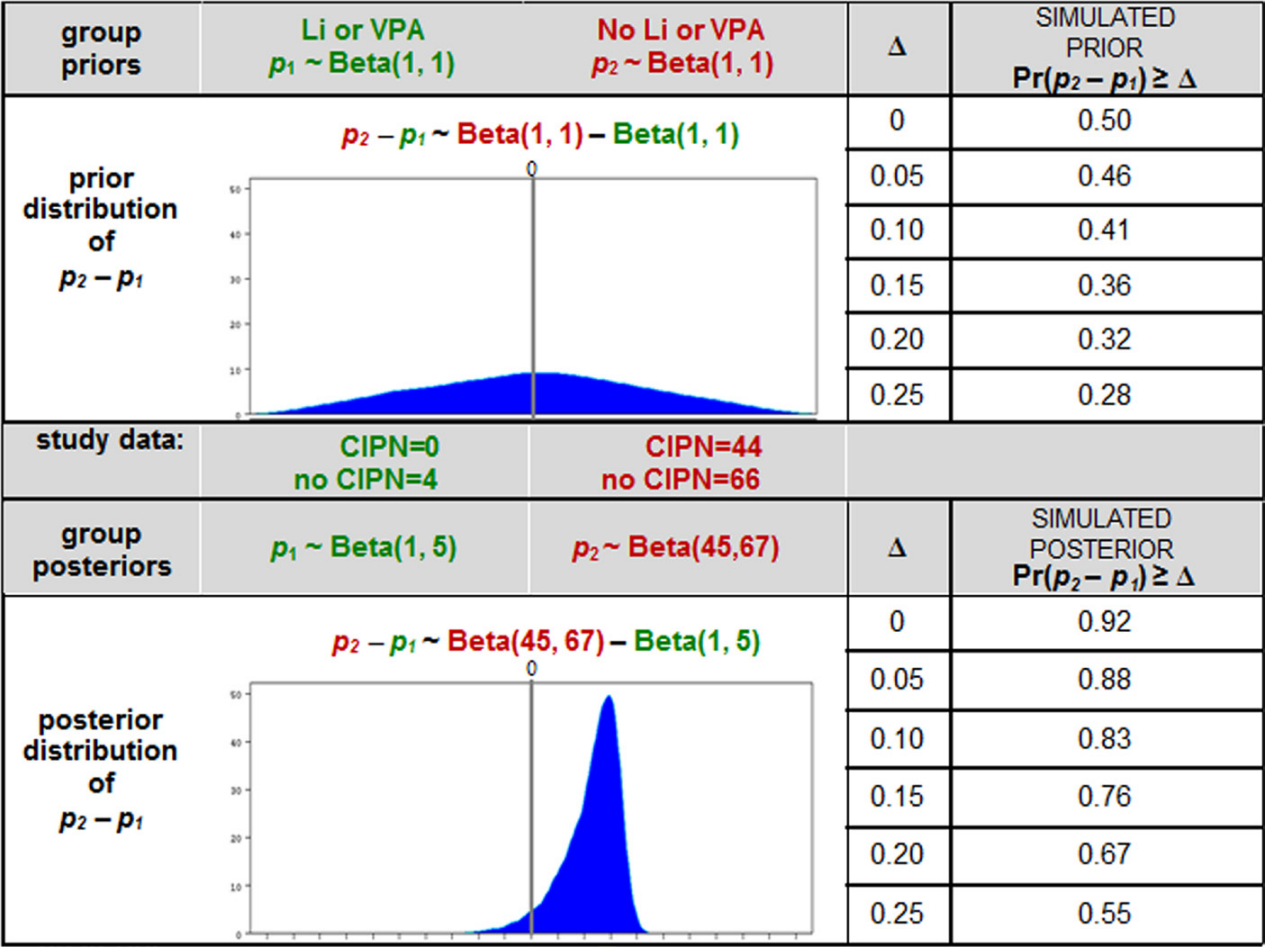
This table shows a comparison of: (i) the completely uninformed prior belief regarding *p*_*2*_ – *p*_*1*_ when the non-informative prior for each of *p*_*1*_ and *p*_*2*_ is Beta(1, 1) versus (ii) the posterior belief regarding *p*_*2*_ – *p*_*1*_ when the priors have been updated based on the observed data For example, our prior belief regarding the probability that *p*_*2*_ *– p*_*1*_ > 0 was 50%; our updated belief based on our data is now 92%. Similarly, our prior belief regarding the probability that *p*_*2*_ *– p*_*1*_ > 0.20 was 32%; our updated belief incorporating our data is now 67%.

The Bayesian approach considers new data as information that can be used to update prior beliefs and to interpret signals. It is well documented that a Bayesian approach (with prior distributions chosen with thoughtful and careful analytic consideration) is especially more appropriate for signal detection than a frequentist approach when some types of patients are rare and sample sizes are small [15–17]. The data from the 4 patients with exposure to Li or VPA and the 110 without that exposure is valuable new information that we did not have prior to the chart review and from which we can learn. We were interested in using these data to (i) elucidate a likely range (via probability distribution) for the magnitude of the Li or VPA effect on CIPN incidence, in order to (ii) assess the prudence of moving forward to a RCT, and (iii) to inform the design of the RCT. The logic of our Bayesian analysis was as follows: Prior to obtaining the chart review data, we conservatively consider ourselves completely uninformed (i.e. chose a ‘non-informative Beta(1,1) prior distribution’) regarding the proportion of incident CIPN cases in the patients who had been taking Li or VPA (*p*_*1*_), as well as those who had not been taking Li or VPA (*p*_*2*_), and derived the prior distribution for the difference of these proportions *p*_*2*_*–p*_*1*_ in the population from which our patients were selected. After examining the data, we used our new information (i.e. 0 CIPN of 4 in the “*Li or VPA*” group and 44 CIPN of 110 in the “*No Li or VPA”* group) to mathematically update the prior probability distributions to the respective posterior probability distributions for the group CIPN proportions: *p*_*1*_∼Beta(1, 5) and *p*_*2*_∼Beta(45, 67). The mathematics for deriving the posterior Beta distributions is discussed elsewhere [18]. These probability distributions for *p*_*1*_ and *p*_*2*_ are represented by the red and blue curves, respectively, in Figure 2. Thus, the probability distribution for the treatment effect *p*_*2*_ *– p*_*1*_ is the difference Beta(45, 67)–Beta(1, 5) (Figure 3). Based on this posterior distribution for *p*_*2*_ *– p*_*1*_, we updated our belief from 50% to 92% for the probability that *p*_*2*_ *– p*_*1*_ > 0, and from 32% to 67% for the probability that Li or VPA reduces the CIPN proportion by at least 0.20, i.e. *p*_*2*_ *– p*_*1*_ > 0.20 (Figure 4).

### Frequentist analysis

We test the null hypothesis *H*_*0*_*: the “Li or VPA” treatment effect p*_*2*_ *– p*_*1*_ *= 0* against the one-sided alternative hypothesis *H*_*1*_*: the “Li or VPA” treatment effect p*_*2*_ *– p*_*1*_ *> 0*. Results of Fisher’s exact test did not show a statistically significant difference, with one-sided *p*-value = 0.1577. For the difference *p*_*2*_ *– p*_*1*_ = 0.40, the exact one-sided 90% and 95% confidence intervals are (–1, 0.46) and (–1, 0.48), respectively. The exact two-sided 90% and 95% confidence intervals are (–0.06, 0.48) and (–0.15, 0.50), respectively [19].

## DISCUSSION

In this study we used retrospective data from patients treated for cancer to obtain preliminary evidence as to whether patients concurrently treated with chemotherapy and with Li or VPA experienced CIPN at a lower rate than patients who received chemotherapy alone. Previously, using a variety of biophysical and biochemical assays, we found a novel calcium binding protein, neuronal calcium sensor 1 (NCS1), that binds to taxanes and vinca alkaloids [10, 11]. When these chemotherapeutic drugs bind to NCS1, a pathological cascade leading to CIPN is initiated [10, 11, 14]. We found that low-doses of Li or VPA, agents commonly used to treat psychiatric and neurologic disorders with a well understood safety profile, inhibit the actions of NCS1 [20] and prevent CIPN in animal models [14]. The present study uses retrospective clinical information extracted from patients’ charts to examine whether the results from the xenograft model [14] are translatable to human subjects and whether further human studies, such as a randomized controlled clinical trial, should be pursued. We chose the time period for our observational study to be between 2007 and 2013 due to the fact that the use of taxanes was less common for certain cancers prior to 2007, e.g. prostate cancer or esophageal/gastric cancer, and the fact that Li and VPA have been increasingly replaced by newer generations of psychotropic medications in recent years. Due to these limitations we identified only 4 patients who were concurrently receiving the drugs of interest. Nevertheless, these 4 patients did provide useful information which yielded a preliminary signal that our data supports further research investigating the role of Li and VPA as preventive agents for CIPN.

As expected in a patient population with a high level of comorbidities, many patients had pre-existing neuropathy (20 out of 114 patients with two or more chemotherapy cycles), probably related to diabetes mellitus and/or excessive alcohol consumption. Otherwise, patients were fairly heterogeneous in terms of underlying disease. While we are unable to confirm compliance with oral medications such as Li, VPA, TCA, SSRI and SNRI with absolute certainty, only patients who had active outpatient prescriptions that were being filled on a regular basis according to the electronic pharmacy records were considered to be on active concurrent treatment with these agents while receiving chemotherapy. We included patients taking other psychoactive medications (TCA, SSRI, SNRI) which are more commonly prescribed in the modern era, however, use of these drugs was not noted to have any protective effect from development of neuropathy.

In the frequentist analysis of the data, statistical significance (*p* = 0.1577) could not be achieved for a hypothesis of a difference greater than 0 for the proportion of patients on chemotherapy alone who developed CIPN versus those patients who were receiving Li or VPA prior to initiating chemotherapy. Bayesian analysis does not restrict us to testing a specific “*Li or VPA”* effect size (e.g. 0); rather, it provides a probability distribution for the possible treatment effect sizes for *p*_*2*_ *– p*_*1*_, and displays the location of the range of most likely values for *p*_*2*_ *– p*_*1*_. Prior to collecting and analyzing the data, our ‘uninformed’ belief regarding the odds of which group had the smaller CIPN rate, those exposed to “*Li or VPA”* or those not exposed, was ‘50:50’. From the data, we learned that we can update our belief regarding these probabilities to ’92:8’ in favor of “*Li or VPA”*. Among other learnings, we now believe that there is a 67% probability that “*Li or VPA”* reduces the CIPN proportion by more than 0.20 compared with those not exposed to Li or VPA, i.e. *p*_*2*_ *– p*_*1*_ > 0.20 (Figure 4). Regardless of the statistical analytic method, and despite the small number of patients treated with Li or VPA in the sample, it is remarkable that the “*Li or VPA”* group did not have any reported cases of CIPN at all.

A challenge to the validity of findings from data obtained in non-randomized observational studies is the possibility that the findings are inherently biased (e.g. treatment selection bias). One method for potentially mitigating some estimation bias is to use ‘propensity scoring’ methods to identify patients in each group who are similar in their propensity (beyond a psychiatric diagnosis) to be receiving Li or VPA treatment [21]. Although the full analysis is not presented here, we used logistic regression to estimate each patient’s probability of being on Li or VPA as a function of aforementioned covariates. There were 64 patients in the “*No Li or VPA”* group with similar propensities to be treated with Li or VPA as those in the “*Li or VPA”* group. We compared these patients with regard to CIPN outcome, having adjusted for the propensity to be on Li or VPA, and found results very similar and substantively identical to those reported above.

A Bayesian approach to data analysis and interpretation (e.g. posterior probability distribution) is not as common in the literature as the frequentist approach (e.g. hypothesis testing, *p*-values, and confidence intervals). However, software development over the past few decades has made Bayesian methods more computationally accessible to more researchers, and advantages over frequentist limitations have made it more attractive and practical [22].

In the case of a small sample size, investigators may avoid conducting studies or analyzing valuable hard-to-find information when there is insufficient power or precision to obtain good estimates or meaningful results using frequentist methods [15]. Frequentist methods are typically calculated by assuming large sample approximations (i.e., the central limit theorem) or by applying low-powered non-parametric methods. Bayesian statistics is based on posterior probability distributions computed using Markov chain Monte Carlo (MCMC) procedures, and is not based on large sample theory. Thus large samples are not required for meaningful, valid, and useful interpretation of the evidence at hand [16].

As a result, there has recently been burgeoning visibility for Bayesian methods: inclusion of Bayesian computation in standard statistical software packages (e.g. SAS^®^ [23, 24], Stata^®^, SPSS, R, Mplus), increased inclusion of Bayesian methods in curriculum for researchers [25], increasing rate of publication of books and manuscripts using Bayesian methods [26], and sanctioning by key scientific entities (e.g. FDA, EMEA) [22, 27–29]. In this paper, we provide an example of an analysis for which the frequentist approach is not appropriate, whereas Bayesian methods enabled us to use our data to inform a decision regarding the utility of future research.

Li and VPA are both FDA approved medications that have well-established safety profiles; most of the adverse effects associated with Li and VPA such as cardiac arrhythmias, central nervous toxicities and hepatotoxicity are associated with higher doses. Our prior animal model work used lower doses of Li and VPA than those used as treatment doses for bipolar disorder. We are hopeful that this study will allow us to examine the use of lower dose Li or VPA in prevention of CIPN. The NCS1 pathway represents a newly identified molecular cascade that explains taxane and vinca alkaloid-induced neurological impairment. Agents that interfere with the NCS1-dependent pathway may offer protection from changes in neurological functions in patients with a variety of cancers, including breast, ovarian, lung, and prostate cancers. Our results presented here suggest that concurrent use of Li or VPA with chemotherapeutic treatment regimens could reduce the risk of CIPN and therefore greatly improve patients’ quality of life during and after chemotherapy. This work forms the initial support for the search for future therapies that will decrease side effects, will greatly improve quality of life following chemotherapy, and potentially increase overall patient survival because fewer patients will drop out of treatment due to adverse effects.

## METHODS

### Chart review

Our study was a retrospective chart review at the Veterans Affairs (VA) Connecticut Healthcare System following institutional guidelines and procedures approved by the Human Investigation Subcommittee of the VA Connecticut Healthcare System. No consent from individual patients was required as the data were analyzed anonymously. Patients who never underwent prior chemotherapy and received first-line taxanes with or without platinum agents chemotherapy from January 1, 2007 until December 31, 2013 were identified via the cancer registry and pharmacy records. Patients with multiple active cancer diagnoses were excluded. A total of 135 cases with one cancer diagnosis who had received docetaxel chemotherapy were identified (Figure 1). Of the 135 cases, 54 received docetaxel chemotherapy only, without any exposure to platinum chemotherapy and no other taxane exposure; 75 received taxanes in sequence or concurrently with platinum chemotherapy and 24 patients received paclitaxel and docetaxel sequentially. All patients who received both paclitaxel and docetaxel also had platinum chemotherapy. 21 were excluded from the study as they had received less than 2 cycles of docetaxel chemotherapy and therefore their risk of developing CIPN was low. Of the remaining 114 cases, 4 patients were on concurrent Li or VPA while receiving chemotherapy.

Pharmacy records in the electronic medical record were reviewed to determine if the subjects had an active prescription for Li, VPA, tricyclic antidepressants (TCA), selective serotonin reuptake inhibitors (SSRI) or selective norepinephrine reuptake inhibitors (SNRI) while receiving concurrent chemotherapy. Concurrent treatment was defined as being started on the medication of interest at least 1 month prior to initiation of chemotherapy and continuing on the drug during the duration of chemotherapy treatment. Notes were systematically analyzed to determine if patients had pre-existing neuropathy or if they developed new or worsening neuropathy during the course of chemotherapy treatment or within 6 months after completion of chemotherapy. Data were also collected on patients’ age, cancer type and stage, type and duration of chemotherapy, pre-existing neuropathy and comorbidities.

### Statistical analytic methods

Because we were examining evidence from a preliminary observational study for the purpose of signal detection, and not formally testing a hypothesis using data from a clinical trial, we used a Bayesian approach to interpret the signal from the data as our primary analysis [18]. Specifically, we defined the “*Li or VPA”* treatment effect as the difference between the proportion of CIPN cases among the “*No Li or VPA”* patients (*p*_*2*_) and that of the patients on “*Li or VPA”* (*p*_*1*_). For the Bayesian analysis, we used a non-informative Beta(1,1) prior probability distribution for both *p*_*1*_ and *p*_*2*_ to represent our ‘uninformed’ baseline knowledge of the proportions of CIPN cases in each group. To simulate the prior and posterior probability distributions of *p*_*2*_ *– p*_*1*_, we generated 15,000 random draws from the difference of the two Beta probability distributions using PASS15 software [30]. From the posterior distribution of *p*_*2*_ *– p*_*1*_, we can assess the probability associated with a positive treatment effect signal. For comparison with a frequentist analysis, Fisher’s exact test was performed to test *p*_*2*_ *– p*_*1*_ > 0 using the *FREQ* procedure in SAS^®^ v9.4 (SAS/STAT v13.1) [31], and exact confidence intervals were calculated for *p*_*2*_ *– p*_*1*_ using the R package *ExactCIdiff* [19].
